# Potentiation of Gamma Aminobutyric Acid Receptors (GABA_A_R) by Ethanol: How Are Inhibitory Receptors Affected?

**DOI:** 10.3389/fncel.2016.00114

**Published:** 2016-05-06

**Authors:** Benjamin Förstera, Patricio A. Castro, Gustavo Moraga-Cid, Luis G. Aguayo

**Affiliations:** ^1^Laboratory of Neurophysiology, Department of Physiology, University of ConcepcionConcepcion, Chile; ^2^Laboratory of Environmental Neurotoxicology, Department of Biomedical Sciences, Faculty of Medicine, Universidad Católica del NorteCoquimbo, Chile; ^3^Hindbrain Integrative Neurobiology Laboratory, Institut de Neurobiologie Alfred FessardGif-Sur-Yvette, France

**Keywords:** alcoholism, ethanol, GABA, GABA_A_R, GlyR

## Abstract

In recent years there has been an increase in the understanding of ethanol actions on the type A γ-aminobutyric acid chloride channel (GABA_A_R), a member of the pentameric ligand gated ion channels (pLGICs). However, the mechanism by which ethanol potentiates the complex is still not fully understood and a number of publications have shown contradictory results. Thus many questions still remain unresolved requiring further studies for a better comprehension of this effect. The present review concentrates on the involvement of GABA_A_R in the acute actions of ethanol and specifically focuses on the immediate, direct or indirect, synaptic and extra-synaptic modulatory effects. To elaborate on the immediate, direct modulation of GABA_A_R by acute ethanol exposure, electrophysiological studies investigating the importance of different subunits, and data from receptor mutants will be examined. We will also discuss the nature of the putative binding sites for ethanol based on structural data obtained from other members of the pLGICs family. Finally, we will briefly highlight the glycine gated chloride channel (GlyR), another member of the pLGIC family, as a suitable target for the development of new pharmacological tools.

## Introduction

Alcohol is a potent depressant drug and one of the most commonly used mind altering substance worldwide. The acute and chronic effects of alcohol abuse cause large medical, economic and social burdens. It is widely accepted that ethanol acts in the central nervous system (CNS) in a considerably complex manner as it affects several receptor-ion channel complexes such as the γ–aminobutyric acid type A receptor (GABA_A_R), glycine receptor (GlyR) and N-methyl-D-aspartate receptor (NMDAR) by different mechanisms that can be either direct (binding) or indirect (kinases, G proteins). This level of complexity presents a high threshold to overcome for the successful development of pharmacological tools to assist behavioral intervention in chronic alcohol abuse and treatment of life-threatening acute effects of excessive drinking. The therapeutic options that are currently available present limited efficacy, lack of adherence and serious side effects (Liang and Olsen, 2014; Spanagel et al., 2014). Therefore, it is crucial to further elucidate the mechanisms of action of ethanol in the CNS in order to meet the challenge of developing more specific and effective therapeutics. As the main inhibitory pentameric ligand gated ion channel (pLGIC) in the brain and one of the major targets of ethanol modulation, the GABA_A_R is one of the principal targets for pharmacotherapy. The evidence for the GABAmimetic effects of ethanol further supports the relevance of GABA_A_R. GABA_A_R can be potentiated by low to intermediate concentrations of ethanol (5–50 mM, corresponding to a few drinks up to complete intoxication). In recent years there has been an increase in the understanding of ethanol effects on GABA_A_R, however, the mechanism of action, whether it is direct or indirect, allosteric or not and which assembly of subunits are influenced in what way, is still not fully understood. In fact, a number of publications have shown differing results, and thus several fundamental questions on the mechanism of action still remain unanswered. The resolution of these questions will provide a more definitive picture on which GABA_A_R subunits are affected by ethanol in what way and in which regions of the CNS. This review aims to help put aside the conflicting available results and think about new key studies that will help answer these questions. For example, focusing the studies on extra-synaptic rather than on synaptic GABA_A_Rs.

The present review will focus on the involvement of GABA_A_R in the effects of alcohol and specifically the modulation of GABA_A_R by acute ethanol exposure. We will address a body of data linking GABA_A_R to ethanol effects and give an overview of evidence from human genetic studies and ethanol response in animal models, the effects of pharmacological compounds on the response to ethanol and the brain regions investigated in this context. Changes to the GABAergic system after chronic exposure to ethanol will be shortly reviewed and discussed in relationship to the acute effects. We will then focus on the immediate, direct and indirect, synaptic and extra or non-synaptic effects of ethanol on GABA_A_R. To elaborate on the immediate, direct modulation of postsynaptic GABA_A_R by acute ethanol, electrophysiological evidence from studies with different subunits, data from receptor mutants and structural information such as binding studies will be discussed. Presynaptic effects of ethanol will be mentioned briefly and we would like to refer the reader to the excellent recent review on this topic (Kelm et al., 2011). Finally, we will briefly highlight the glycine gated chloride channel (GlyR), another inhibitory member of the pLGIC family, as a more suitable target for the development of new pharmacological tools.

## Functions and Distribution of GABA_A_R Subunits in Comparison to GABA_B_R and GABA_C_R

Three types of GABAR have been described up to now, two of those, the GABA_A_R and the GABA_C_R are ligand gated ionic channels (LGIC), whereas GABA_B_R is a G-protein coupled receptor (GPCR). Extensive research has been focused on the study of the functions, diversity and locations of GABA_A_R describing 19 subunits (α1–6, β1–3, γ1–3, δ, ε, π, ρ1–3 and θ). These receptors are broadly expressed in the CNS representing about 20% of the synapses in cortex, hippocampus, thalamus and cerebellum, where they possibly control cognitive functions such as memory, language and attention (Mody et al., 1994; Michels and Moss, 2007). Different receptor subunit composition has been shown of which the principal representation corresponds to: α1β2γ2 (43%), α2β2/3γ2 (18%), α3βnγ2/3 (17%), α2βnγ1 (8%), α5β3γ2/γ3 (4%), α6βγ2, α6βδ, α4βδ and others (2%). Also, receptors containing γ have been defined as synaptic while receptors containing α4, α5, α6 and δ are largely extra or non-synaptic (McKernan and Whiting, 1996; Cherubini and Conti, 2001). Furthermore, GABA_B_R activation at presynaptic locations suppresses neurotransmitter (NT) release by inhibition of voltage sensitive Ca^2+−^-channels, while postsynaptic receptors induce a slow inhibitory postsynaptic current by gating KIR_3_-type K^+^-channels, which hyperpolarizes the membrane and shunts excitatory current (Bettler and Tiao, 2006). Two subtypes of GABA_B_R have been described (B1 and B2), each featuring 2 isoforms (B1a, B1b and B2a and B2b) that are located pre- and post-synaptically and found in almost all brain neuronal populations (Bettler and Tiao, 2006). GABA_C_R is composed of three subunits (ρ1–3) that originally belonged to the GABA_A_R subtype and has been associated to visual processing, regulation of sleep–waking rhythms, pain perception, memory, learning, regulation of hormones and neuroendocrine gastrointestinal secretion. These receptors are localized in the retina, thalamus, hippocampus, pituitary and gastro intestinal tract (Chebib, 2004). The potentiation mediated by ethanol is restricted to GABA_A_R.

## Evidence for a GABAmimetic Action of Ethanol on the Central Nervous System

It is known that GABA_A_R are highly expressed throughout the brain, i.e., cerebellum, hippocampus, cerebral cortex, amygdala, VTA, nucleus accumbens and thalamus (Pirker et al., 2000; Schwarzer et al., 2001; for review see Faingold et al., 1998; Mihic, 1999). These areas of GABA_A_R expression represent potential sites for the GABAmimetic effect of ethanol. So far, most brain regions examined have shown similar ethanol actions at intoxicating concentrations. A concentration of 100–200 mM ethanol represents severe alcohol toxicity and is likely to be lethal (Lovinger and Homanics, 2007). Therefore, while used in some studies, these ranges of concentrations are unlikely to occur *in vivo* under physiological conditions. Some laboratories report significant potentiation of γ subunit-containing receptors with ethanol concentrations as low as 50 mM, but these concentrations are still equivalent to heavy intoxication (Ueno et al., 2001). For comparison, consuming a single drink on average results in a blood concentration corresponding to roughly 5 mM and already elicits mood changes, impaired cognition and reduced motor coordination, 20 mM would cause a strong intoxication, and a blood concentration corresponding to 100 mM will kill most individuals. The legal limit of blood ethanol concentration for drivers in many countries is 17 mM (Lovinger and Homanics, 2007). Consequently, relevant studies should always consider a range of ethanol concentration from low to high, starting from a concentration as low as 5–10 mM and testing intermediate and high concentrations up to 100 mM, to be of pharmacological interest.

As described later, a body of genetic and transgenic evidence supports the involvement of GABA_A_R in the behavioral response to ethanol (see Tables 1, 2). Additional supporting data comes from the effects that pharmacological substances interacting directly with GABA_A_R have on the response to ethanol. This notion is also supported by the similarities between the sedative and inhibitory effects of ethanol and drugs known to act on GABA_A_R, such as benzodiazepines and general anesthetics (Grobin et al., 1998; Breese et al., 2006). Pharmacological studies using drug discrimination choice support this GABAmimetic action of ethanol (Grant, 1999). Furthermore, it was shown that negative modulators of GABA_A_R reduced alcohol intake in animal models (Wegelius et al., 1993). Overall, these types of studies are strongly supportive of GABAergic actions for a range of ethanol concentrations.

**Table 1:** **Genetic evidence linking GABA_A_R to human alcoholism**.

Study	Affected alcohol related behavior
*GABRA2* Human SNP	Increased responses to alcohol related cues in reward centers of the brain (Kareken et al., 2010; Villafuerte et al., 2012); changes in hedonic value of alcohol (Haughey et al., 2008) and subjective response to ethanol (Pierucci-Lagha et al., 2005; Haughey et al., 2008)
*GABRG1* Human polymorphism	Linked to alcoholism independently of the impact of *GABRA2* (Covault et al., 2008; Enoch et al., 2009)
*GABRA6* Human association	Significant genome association with human alcoholism (Li et al., 2014)
*GABRG2* Human association	Significant genome association with human alcoholism (Li et al., 2014)
*GABRR1 and GABRR2*	Significant genome association with
Human association	human alcoholism (Xuei et al., 2010)

**Table 2:** **Genetic animal models linking behavioral alterations to GABA_A_R**.

Model or Study	Affected alcohol related behavior
*GABRA1* Mouse allelic noncoding variation*	Ethanol induced hypothermia and motor incoordination, ethanol-conditioned taste aversion and acute ethanol withdrawal (Ueno et al., 2001)
GABA_A_R **α**1 ethanol insensitive Mouse KI	Reduced sedative and increased anxiolytic effect of ethanol (Lobo and Harris, 2008)
GABA_A_R **α**2(S270H/L277A) ethanol insensitive Mouse KI	No taste aversion and hyperlocomotion, decreased hypnosis but still anxiolytic (Blednov et al., 2011)
GABA_A_R **α**2 Mouse KO	Reduction of conditioned taste aversion and faster recovery of ethanol induced incoordination (Blednov et al., 2013)
GABA_A_R **α**3 Mouse KO	Prolonged motor-incoordination (Blednov et al., 2013)
GABA_A_R **α**6 Mouse KO	Effect of ethanol unchanged, possibly due to compensatory adaptations (Homanics et al., 1997, 1998)
*GABRA6* Mouse allelic non-coding variation*	Ethanol-induced hypothermia and motor incoordination, ethanol-conditioned taste aversion and acute ethanol withdrawal (Ueno et al., 2001)
*GABRA6* ethanol sensitive rat allelic non-coding variation**	Increased motor impairment (Eriksson and Sarviharju, 1984; Korpi et al., 1993)
*GABRA6* Ethanol sensitive rat R100Q SNP	Increased ethanol sensitivity in these rats seems to be independent of R100Q (Botta et al., 2007), despite previous suggestions (Korpi et al., 1993; Hanchar et al., 2005)
GABA_A_R **β**1 L285R mutant Mouse	Increased ethanol consumption without change in ethanol potentiation (Anstee et al., 2013)
GABA_A_R **β**3 N265M mutant Mouse KI	Increased tolerance and withdrawal but little change in acute ethanol sensitivity (Sanchis-Segura et al., 2007)
GABA_A_R **α**2 Mouse KO	Reduced anticonvulsant effects, ethanol consumption and withdrawal (Mihalek et al., 2001)
*GABRG2* Mouse allelic coding variation*	Ethanol-induced hypothermia and motor incoordination, ethanol-conditioned taste aversion and acute ethanol withdrawal (Buck et al., 1997)
GABA_A_R **γ**2L Mouse KO	Normal ethanol response (Homanics et al., 1999)
GABA_A_R ρ Mouse KO	Reduced ethanol consumption and faster recovery of ethanol induced incoordination (Blednov et al., 2003)
PKCε Mouse KO	Increased hyperlocomotion and sedation, reduced voluntary alcohol consume (Hodge et al., 1999; Olive et al., 2000), increased alcohol aversion (Newton and Messing, 2007) and increased response to direct GABA_A_R antagonist muscimol (Ueno et al., 2001)
PKC**δ** Mouse KO	Resistance to ethanol intoxication and reduced or abolished potentiation of tonic GABAergic currents (Choi et al., 2008)
PKC**γ** Mouse KO	Shorter loss of righting reflex and less sensitive to ethanol-induced hypothermia (Harris et al., 1995)

*Either of these polymorphisms may account for the changes (Buck et al., 1997; Ueno et al., 2001). **Other co-segregated factors may account for the changes (Congeddu et al., 2003; Radcliffe et al., 2004).

**Figure 1:**
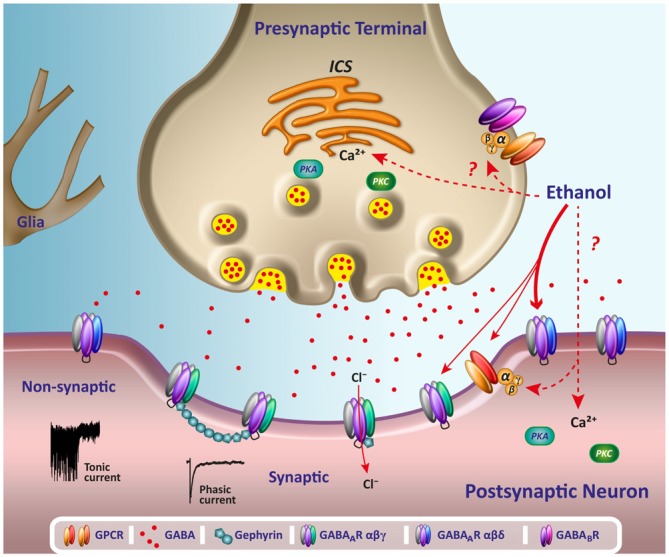
**Acute effects of ethanol on GABAergic transmission.** The scheme illustrates several potential acute pre and postsynaptic sites for the effects of ethanol on GABAergic neurotransmission. Reported changes on the release of presynaptic GABA might be mediated by changes on calcium release from intracellular calcium stores (ICS) following activation of G-protein coupled receptors (GPCR) or phosphorylation by protein kinase A (PKA) and C (PKC). Changes on the activation of GPCR, such as GABA_B_R, could affect GABA release and alter the tonic Cl^−^ current associated to non-synaptic GABA_A_R δ containing receptors through spillover of synaptically released GABA. At postsynaptic domains, acute low ethanol concentrations of alcohol appear to modulate primarily non-synaptic GABA_A_R (see thicker arrow) by a mechanism that might involve direct binding to the general anesthetics site of action or by intracellular signaling pathways, G protein, PKC and PKA or calcium release. The scheme also shows representative traces of a phasic current, activated by synaptic receptors, and a sustained small desensitizing tonic current, mediated by non synaptic receptors.

Cerebellar neurons have been critical for the study of ethanol effects on GABA_A_R. In the 90’s, *in vivo* recordings showed that ethanol depressed Purkinje neuron firing in rats, and this depression was antagonized by a benzodiazepine partial inverse agonist (RO15–4513), as well as by the competitive GABA_A_R antagonist bicuculline (Palmer et al., 1988; Palmer and Hoffer, 1990). Moreover, GABA-induced inhibition of Purkinje cell firing was enhanced by acute ethanol administration and most interestingly, it was reported that these cells were sensitized to the effects of ethanol by activation of β-adrenergic receptors, which suggests that the action of ethanol on GABA_A_R in these neurons could be affected by G protein activation (Lin et al., 1994; Freund and Palmer, 1997; Yang et al., 1998). The notion that G proteins are involved in mediating effects of ethanol is supported by other experiments showing that the potentiation of GABA_A_R in hippocampal neurons was dependent on the presence of guanine nucleotides that can affect the activation of G proteins (Weiner et al., 1997). A body of information of pre- and postsynaptic effects of ethanol is summarized in Figure 1.

Low doses of ethanol were found to increase the frequency of spontaneous inhibitory post-synaptic currents (sIPSCs), and miniature inhibitory post-synaptic currents (mIPSCs) at higher doses, in synapses of cerebellar Golgi and granule cells (Carta et al., 2004). Additionally, local manipulation of GABA_A_R activity in distinct brain regions can have different effects on ethanol responses, i.e., reduction in GABA_A_R activity in the VTA reduces alcohol intake, but the same effect can be achieved by increased GABA_A_R activity in the nucleus accumbens (Vengeliene et al., 2008). A number of laboratories demonstrated that ethanol potentiates GABA_A_R-mediated synaptic transmission via an increase in GABA release from presynaptic terminals in several brain regions including the cerebellar cortex (Valenzuela and Jotty, 2015). Additionally, tonic currents associated with the activation of non-synaptic GABA_A_R in granule cells were found to be increased, but this effect was dependent on action potentials and therefore most likely mediated by the spill-over of synaptically released GABA (Carta et al., 2004; Siggins et al., 2005; Weiner and Valenzuela, 2006).

In terms of molecular characteristics for the potentiation of GABA_A_R, it is known that the ethanol sensitive receptor containing extra-synaptic δ-subunit responsible for the tonic current is expressed in the nucleus accumbens, hippocampus, thalamus, cortex and cerebellum (Pirker et al., 2000; Brickley et al., 2001; Schwarzer et al., 2001; Stell et al., 2003; Cope et al., 2005; Jia et al., 2005; Chandra et al., 2006). Interestingly, the knock down of GABA_A_R δ expression by RNAi in the dorsomedial shell region of the nucleus accumbens in mice caused a reduction in ethanol preference, probably by disinhibition of neuronal targets in the VTA, ventral pallidum or lateral hypothalamus (Nie et al., 2011; Tabakoff and Hoffman, 2013). Another line of support for involvement of the δ subunit comes from the use of a partial agonist of the benzodiazepine receptor, RO15–4513, that has high affinity for several GABA_A_R and acts more selectively on extrasynaptic α6/β containing receptors at the benzodiazepine binding site (Lüddens et al., 1990; Korpi et al., 1993, 1999). Its administration reduced voluntary ethanol intake, anxiolytic behavior and motor impairment, although clinical application was abolished after finding that it also increased withdrawal seizures (Suzdak et al., 1986a; Hoffman et al., 1987; Becker and Hale, 1991; June et al., 1991; Schmitt et al., 2002; Lovinger and Homanics, 2007). Also, application of the structural GABA analog Gabapentin in the central amygdala reversed alcohol operant behavior and increased IPSC amplitude in alcohol dependent rats, but had the opposite effect in naïve rats, and the effect on current amplitude was abolished by application of GABA_B_R blockers (Roberto et al., 2008). Therefore, the effects of chronic alcohol exposure also need to be taken into consideration when evaluating acute effects.

The direct GABA_B_R agonist baclofen reduced alcohol intake in mice, although it showed limited effects in human subjects (Colombo et al., 2004; Maccioni and Colombo, 2009; Tyacke et al., 2010; Addolorato et al., 2011, 2012; Brennan et al., 2013). GABA release in amygdala, VTA and hippocampus at high concentrations of ethanol appears to rely on activation of GPCRs, and the activation of the metabotropic GABA_B_R can attenuate GABA release (Peris et al., 1997; Nie et al., 2004, 2009; Wu et al., 2005; Silberman et al., 2009; Kelm et al., 2011). Thus, the GABA_B_R agonist GHB which also acts as a partial agonist for δ subunit containing GABA_A_R might affect alcohol intake by complex inhibitory mechanisms (Absalom et al., 2012). Additionally, increased expression of extra synaptic recombinant GABA_A_R containing α6 and δ subunits was positively modulated by alcohol (for review see Wallner et al., 2006).

## Human and Animal Genetic Studies Support Effects of Ethanol on GABA_A_Rs

If GABA_A_Rs are important for alcohol use disorders (AUD), one would expect that gene variations would have an impact on alcohol abuse behaviors. In agreement with this idea, human genetic studies indicated a central role of GABA_A_R in alcoholism (Engin et al., 2012; see Table 1). For example *GABRA2*, the gene encoding the GABA_A_R α2 subunit, features small nucleotide polymorphisms (SNP) which do not affect the coding sequence, but correlate with mRNA levels in post-mortem prefrontal cortex (Haughey et al., 2008) of alcoholic patients. These SNPs were linked to alcohol dependence, specifically increased responses to alcohol related cues in reward centers of the brain, the hedonic value of alcohol, and subjective responses to ethanol in humans (Edenberg et al., 2004; Pierucci-Lagha et al., 2005; Haughey et al., 2008; Kareken et al., 2010; Villafuerte et al., 2012). Reduced expression of the α2 subunit in humans might have an effect similar to the α2 null mutant in mice discussed below, facilitating alcohol abuse by diminishing some of its negative side effects. In addition, *GABRA6*, *GABRG1*, and *GABRG2* polymorphisms (GABA_A_R α6, γ1 and γ2 subunit) appear to be linked to alcoholism independent of *GABRA2* (Covault et al., 2008; Enoch et al., 2009; Li et al., 2014). Furthermore, the genes coding for GABA_A/C_R Rρ1 and 2 were also implicated in human alcohol dependence (Xuei et al., 2010). While these studies suggest involvement of α6, γ1, γ2 subunits and especially α2 subunits in the effects of ethanol, they cannot elucidate the mechanism of action of ethanol.

On the other hand, a correlational analysis study of *GABRG2* allelic variation in the GABA_A_R γ2 subunit in BXD recombinant inbred mouse strains indicated a link between allelic polymorphism in the protein coding sequence and ethanol-induced hypothermia, motor incoordination, ethanol-conditioned taste aversion and withdrawal (Hood and Buck, 2000; Ueno et al., 2001; see Table 2). However, differential expression levels of the GABA_A_R α1 and α6 subunits due to variation in the noncoding sequence of the *GABRA1* and* GABRA6* genes may also be responsible for these ethanol effects in the investigated mouse strains (Ueno et al., 2001). Another naturally occurring polymorphism in the gene coding for the α6 subunit may be responsible for increased motor impairment in selectively bred ethanol-sensitive rats, although it cannot be excluded that other co-segregated factors may account for these differences (Eriksson and Sarviharju, 1984; Korpi et al., 1993; Congeddu et al., 2003; Radcliffe et al., 2004). In agreement to the proposed role of GABA_A/C_R Rρ1 and 2 to alcoholism in humans, ρ1 KO mice show reduced ethanol consumption and faster recovery of ethanol-induced loss of motor coordination (Xuei et al., 2010). Although these studies have provided additional insights into the relative importance of individual subunits for specific effects of ethanol, caution is needed in the interpretation of behavioral responses in transgenic animals with global knock-out of gene products due to possible compensatory mechanisms.

Positive correlations between observations* in vitro* and transgenic mouse lines are not always evident making it difficult to draw conclusions about the role of genes in ethanol behaviors. For example, *in vitro* data from frog oocytes suggested a role of the long splice variant of the GABA_A_R γ2 subunit in the action of ethanol (Wafford et al., 1993), whereas studies in GABA_A_R γ2L knock-out mice, on the other hand, did not show a changed behavioral response to ethanol compared to wild-type mice (Homanics et al., 1999). Also, mutations in the GABA_A_R β3 subunit revealed effects on ethanol tolerance and withdrawal (Sanchis-Segura et al., 2007). In addition, GABA_A_R δ KO mice exhibited reduced anticonvulsant effects of ethanol, lower alcohol consumption and attenuated withdrawal, while other symptoms were not altered, supporting the involvement of extrasynaptic δ/α6 containing receptors in some responses to alcohol. KO of α6, on the contrary, did not show any behavioral changes upon ethanol exposure even though it was accompanied by loss of the δ subunit, indicating the need for α6 in δ subunit assembly (Homanics et al., 1997, 1998; Korpi et al., 1999). These KO mice also showed reduced α1 and β2 expression and exhibited an increased potassium leak current in cerebellar granule cells, which is mediated by activation of TASK-1 and 3 K2P channels (Uusi-Oukari et al., 2000). These changes might be compensatory for the loss of tonic inhibition that is produced by the non-synaptically located subunits (Brickley et al., 2001; Aller et al., 2005). Thus, it is important to consider that the observed differences might be in part due to compensatory adaptations as suggested previously in these transgenic models (Homanics et al., 1997, 1998). On the other hand, a *GABRA6* R100Q SNP related to BDZ-binding properties was linked to increased ethanol sensitivity (Hanchar et al., 2005). However, this allele was found not to be conserved in the investigated line of selectively bred ethanol sensitive rats after 45 generations and further genetic studies did not support an important involvement of the R100Q polymorphism in the observed behavioral response to ethanol (Korpi and Uusi-Oukari, 1992; Crabbe et al., 1999; Radcliffe et al., 2004; Botta et al., 2007). GABA_A_R α2 subunit null mutant mice exhibited a reduction in conditioned taste aversion with ethanol and shorter recovery time from ethanol-induced motor incoordination, while deletion of GABA_A_R α3 led to a slower recovery (Blednov et al., 2013).

The results in GABA_A_R α2 subunit null mutants supporting the involvement of α2 are in agreement with data from α2(S270H/L277A) knock-in mice, which express ethanol insensitive GABA_A_R α2 subunit containing receptors. These KI animals did not develop conditioned taste aversion to ethanol and exhibited a decreased ethanol-induced hypnosis, as well as a complete loss of ethanol-induced motor stimulation, while the anxiolytic properties of ethanol were not abolished (Blednov et al., 2011). While knock-in models might reflect a more physiological condition than knock-out animals, it is important to consider that expression levels may still vary in these mutant animals as compared to wild type. Furthermore, the changes in mutant receptors can affect physiological receptor function. For example, ethanol consumption was increased in a knock-in mouse expressing L285R mutant GABA_A_R β1, although this mutation did not change ethanol potentiation in recombinant mutant receptors *in vitro* (Anstee et al., 2013). Taken together, these studies strongly implicate the α2 subunit in conditioned taste aversion, hypnotic, and motor stimulant effects of ethanol. The knock-in data, in particular, lends strong support to the idea that ethanol potentiation of α2-containing receptors mediates these aspects of ethanol response. The δ KO supports the involvement of GABA_A_R δ in promoting the anticonvulsant effects of ethanol, alcohol consumption and alcohol withdrawal, although KO of α6 does not support these findings. The results for the role of other subunits are even less clear cut. Overall, while these studies suggest a critical involvement of GABA_A_R in the expression of ethanol-induced behavior, they have not provided strong support for a specific molecular involvement in a particular ethanol related behavior. It is also largely unknown if these genotypic modifications on GABA_A_R in human and animals are associated to loss or gain of function, which might have a large impact on behavior, even in the absence of ethanol.

## Studies with Recombinant Receptors Have Not Provided a Clearer Picture

To be able to deal with the large number of possible receptor subunit combinations that might be important for ethanol actions, recombinant receptors have been examined in several expression systems (McKernan and Whiting, 1996; Barnard et al., 1998). Similar to studies in native GABA_A_R, investigation of ethanol effects on recombinant GABA_A_R expressed in frog oocytes have provided conflicting results, in some cases even by the same laboratory (Wafford et al., 1991; Sigel et al., 1993; Marszalec et al., 1994; Mihic et al., 1994; McCool et al., 2003). A study on recombinant receptors in frog oocytes reported that the long γ2L, but not the short γ2S splice variant of GABA_A_R, controlled the ethanol potentiation (Wafford et al., 1991, 1993). Additionally, mutation of the putative phosphorylation S343 site (LLRMFSFK) in the splice insert in the large intracellular loop between TM3 and 4 of the γ2L variant, as well as the administration of kinase inhibitors, both abolished ethanol potentiation (Wafford et al., 1991; Wafford and Whiting, 1992). Thus, *in vitro* results from transiently transfected frog oocytes may not be transferable to mammalian neurons expressing native GABA_A_R, where posttranscriptional processing of the receptors and presynaptic mechanisms may play an additional important role (Harris et al., 1995; Aguayo et al., 2002).

**Figure 2:**
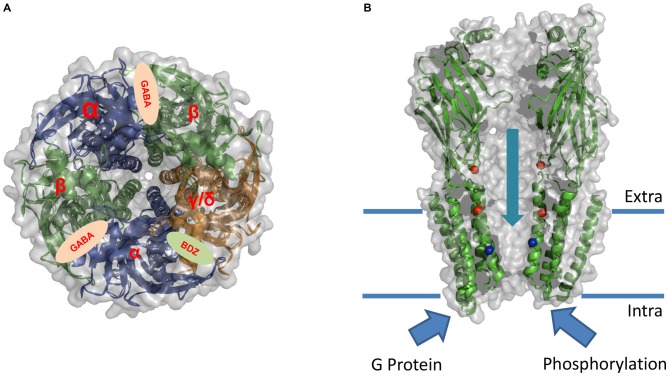
**Sites of action for allosteric modulation of GABA_A_R.** Schematic illustration of the overall architecture and putative modulatory sites of action on GABA_A_R. The model is based in the X-ray structure of GABA_A_R β3 subunit (PDB: 4COF; see Miller and Aricescu, 2014). **(A)** Upper view of the putative subunit stoichiometry and global architecture of the αβγ/δ GABA_A_R. The cartoon shows the binding sites for GABA and Benzodiazepine (BDZ). **(B)** Lateral view showing the suggested binding sites for ethanol (red spheres) and Picrotoxin (blue spheres). This crystal structure lacks the intracellular domain between TM3 and TM4 which was shown to be critical for the modulation of the receptor by G protein and phosphorylation pathways.

In terms of other potentially important subunits for ethanol actions on the receptor, it was reported that while γ subunit containing receptors generally exhibit a low sensitivity to ethanol, GABA_A_R containing δ subunits exhibit a potentiation of up to 50% with ethanol concentrations of 10 mM in oocytes (Wallner et al., 2003, 2006; Lovinger and Homanics, 2007). These results, however, also raised some controversy because other studies did not report potentiation in similar experiments (Borghese et al., 2006; Yamashita et al., 2006). Thus, it is difficult to definitively conclude if the type of subunits that constitutes the GABA_A_R matters or not for the effect of ethanol on the protein complex (see Figure 2).

## Ethanol Binding Sites

While it has been suggested that ethanol can modulate the function of the GABA_A_R using a defined binding pocket (also crucial for the actions of other allosteric drugs), the relative position of the amino acids lining this pocket is still uncertain. So far, two regions of the GABA_A_R have been proposed as critical for ethanol effects: (1) an extracellular binding site located at the α+β3− subunit interface where both the α6R100 and β3Y66 residues contribute to form the ethanol/imidazobenzodiazepine binding site. This site, also important for benzodiazepine effects, is only functional in the α4/6β3δ receptor combination (Wallner et al., 2014); and (2) a binding pocket located in the transmembrane regions formed by residues in the TM2 and TM3 (see Figure 2). In the GlyR and possibly in GABA_A_R, corresponding residues S270 (α2) and S265 (β1) in the TM2 domain and A291 (α2) and M286 (β1) in the TM3 have been suggested to form an ethanol pocket between TM2 and TM3 (Mihic et al., 1997; Mascia et al., 2000; Ueno et al., 2000). Unfortunately, mutagenesis of key residues in the transmembrane regions of the α and β subunits of the GABA_A_R resulted in mutant receptors that displayed dramatic changes in GABA_A_R function, such as loss of function, or spontaneously open ion channels raising the question if the lack of ethanol effects was due to the existence of a site for ethanol or to a change in normal receptor physiology (see Table 3). In addition, these binding sites were not specific for ethanol, but rather water filled cavities able to accommodate a large number of drugs of different physical-chemical properties. On the other hand, comparative experimental data between all members of the pLGICs family showed opposing functional consequences (inhibition v/s potentiation by ethanol), even when the putative pocket-forming residues are conserved. Based on these observations, the hypothesis that the two effects of potentiation and inhibition can coexist in the same receptor gained more recognition in the last years. Molecular studies performed in GABA_A_R, GlyR and nAChR showed that one effect (inhibition or potentiation) could be masked by the other. For instance, the S270I mutation in the GABA_A_R α2 subunit resulted in inhibition by ethanol to a similar magnitude as potentiation in the α2 WT (Mihic et al., 1997). Moreover, changes in the volume of the cavity by the mutation T261W in α2 enhanced the potentiating effects of ethanol in GABA_A_R (Ueno et al., 2000; Johnson et al., 2012). Therefore, these studies suggest the presence of two independent (inhibiting and potentiating) binding sites inside the same receptor (see Figure 2).

**Table 3:** **Effects of GABA_A_R mutations *in vitro***.

Receptor Mutation	Affected receptor functions
GABA_A_R **α**1 S270I TM2 substitution	Not potentiated by 200 mM ethanol* (Mihic et al., 1997; Ueno et al., 1999)
GABA_A_R **α**1**β**1 S265I TM2 substitution	Reduced potentiation by ethanol (Mihic et al., 1997; Ueno et al., 1999)
GABA_A_R **α**2 A291W TM3 substitution	Reduced potentiation by ethanol and spontaneously open state (Mihic et al., 1997; Ueno et al., 1999, 2001)
GABA_A_R **α**2 S265I **β**1 S265I TM2 substitution **γ**2L	Reduced potentiation by 200 mM ethanol (90% WT/15% MUT; Wafford et al., 1991; Wafford and Whiting, 1992; Mihic et al., 1997; Ueno et al., 1999)
GABA_A_R **α**2**β**1 S265I TM2 substitution **γ**2L	Reduced potentiation by 200 mM ethanol (90% WT/20% MUT; Wafford et al., 1991; Wafford and Whiting, 1992; Mihic et al., 1997; Ueno et al., 1999)
GABA_A_R **α**2**β**1 S270I TM2 substitution **γ**2L	Reduced potentiation by 200 mM ethanol (90% WT/34% MUT; Wafford et al., 1991; Wafford and Whiting, 1992; Mihic et al., 1997; Ueno et al., 1999)
GABA_A_R **α**6 R100Q SNP substitution	Increased ethanol response with **βδ** but not **βγ**2 co-expression (Hanchar et al., 2005), but no effect in other studies (Botta et al., 2007)
in BDZ binding site	
GABA_A_R GABA_A_R ρ1 GlyR TM2 to TM3	Potentiated by 200 mM ethanol (88% WT/48% C6; Mihic et al., 1997; Ueno et al., 1999)
45 residue substitution	
GlyR **α**1 GABA_A_R **γ**-loop-2 chimera	No effect on ethanol sensitivity and general receptor function (Perkins et al., 2009)
GlyR **α**1 GABA_A_R **δ**-loop-2 chimera	Increased ethanol sensitivity but no effect on general receptor function (Perkins et al., 2009)
GlyR **α**1 GABA_A_R **δ**-loop-2 A52 chimera	Highly increased ethanol sensitivity but no effect on general receptor function (Perkins et al., 2009)
GlyR **α**1 GABA_A_R **δ**-loop-2 A52E chimera	Reduced ethanol sensitivity (Perkins et al., 2009)
GABA_A_R **γ**2L S265I	Unchanged potentiation by ethanol (Wafford et al., 1991; Wafford and Whiting, 1992; Mihic et al., 1997; Ueno et al., 1999)
GABA_A_R **γ**2L S270I	Unchanged potentiation by ethanol (Wafford et al., 1991; Wafford and Whiting, 1992; Mihic et al., 1997; Ueno et al., 1999)
GABA_A_R **γ**2L TM2/3 loop phosphorylation site mutant	Effect of ethanol blocked (Wafford et al., 1991; Wafford and Whiting, 1992)
GABA_A_R TM2 cysteine residue mutant	Covalent binding of general anesthetics irreversibly increases apparent agonist affinity and prohibits subsequent further potentiation by ethanol (Mascia et al., 2000)

*See also GABA hypersensitive and spontaneously open A2(S270I) mutant (Ueno et al., 2001).

The understanding of the molecular basis for alcohol modulation has been hampered by the lack of ethanol-bound crystal structures for mammalian pLGICs receptors. To date, a major insight on the structural basis of allosteric ethanol sites in pLGICs was the co-crystallization of the prokaryote receptor GLIC bound to ethanol (Sauguet et al., 2014). These crystal structures revealed that GLIC contains at least two different cavities (slightly different to what was previously predicted for mammalian pLGICs) able to accommodate propofol, desflurane and alcohols. The GLIC receptor, however, is normally insensitive to pharmacological concentrations of ethanol (1–100 mM). Introduction of the F14’A mutation inside the ethanol binding site in the pore-lining M2 helix, nevertheless, increased the ethanol sensitivity to similar levels as that observed in mammalian receptors (Howard et al., 2011). Comparison between the wild type and the F14’A mutant structures showed that the absence of the phenylalanine ring expanded the volume of the cavity, allowing the ethanol molecule to enter and stabilize the open conformation of the channel supporting the idea of two opposite binding sites coexisting in the same receptor (Sauguet et al., 2014). A homology model based on the structural data showed a homologous cavity in both GlyR and nAChR, suggesting a common mechanism of modulation in all members of the pLGIC family (Sauguet et al., 2014). In addition, the first X-ray crystal structure of a human β3 GABA_A_R has just been published (Miller and Aricescu, 2014). However, it is not possible to observe an ethanol-binding pocket in this structure perhaps because it is a homopentamer composed of only the β3 subunit, supporting the idea that at least in the GABA_A_R, the putative binding pocket is formed by the interaction of residues from different subunits.

## Effects of Chronic Ethanol Exposure on the GABAergic System

Neuronal cell cultures, as well as animal models, show changes in mRNA levels for different GABA_A_R subunits after alcohol exposure (Kumar et al., 2004; Wafford, 2005). For example, alterations in GABA_A_R subunit expression levels, including changes in the levels of extra-synaptic subunits α6 and δ, have been detected in the cerebellum of rats chronically treated with ethanol (Mhatre and Ticku, 1992; Morrow et al., 1992; Vekovischeva et al., 2000; Sanna et al., 2004; Marutha Ravindran et al., 2007). In neonatal rats, repetitive exposure to ethanol increased expression of the δ subunit in cerebellar granule cells, but the properties of neither tonic nor phasic GABAergic currents were modified (Diaz et al., 2014). Accordingly, either the contribution of GABA_A_R δ is not highly relevant to inhibitory inputs, or compensatory homeostatic mechanisms maintained normal inhibitory input in the treated neurons. Chronic ethanol leads to increased PKCε expression in neuronal cell lines, which in turn stimulates neurite outgrowth and might impact on GABA_A_R function (Messing et al., 1991; Roivainen et al., 1994; Hundle et al., 1995, 1997). Interestingly, animal studies showed that prolonged exposure to alcohol gives rise to chronic effects, some of which countered the acute facilitation of ethanol on GABAergic transmission and could render the brain hyperexcitable during alcohol withdrawal (Diana et al., 2003; Lovinger, 2008; Steffensen et al., 2009; Roberto et al., 2012). Studies in primates and rats have reported that after extended ethanol exposure, expression levels of GABA_A_R α1, 2, 3 and 5 decreased, whereas α4, α6 and γ2 expression increased (Grobin et al., 2000; Cagetti et al., 2003; Anderson et al., 2007). Also, GABA release, IPSC frequency, and amplitude were augmented after chronic ethanol exposure in the central amygdala, while acute ethanol still increased IPSC frequency and amplitude similarly in alcohol naïve and dependent rats (Roberto et al., 2004). Postsynaptically, chronic ethanol seems to also decrease α4 expression in amygdala and nucleus accumbens (Papadeas et al., 2001). Furthermore, altered association of GABA_A_R with PKC changed protein phosphorylation in the cerebral cortex, which was shown to influence clatherin-mediated endocytosis of receptors, changing the ratio of GABA_A_R species that could mediate GABA responses at the cell surface (Kumar et al., 2002; Jacob et al., 2008; Gonzalez et al., 2012). Some of these changes are probably responsible for the changes in animal behavior after chronic ethanol exposure. Changes in subunit expression may for example affect the behaviors affected in corresponding KO models. The decreased expression of GABA_A_R α2 might contribute to the build-up of alcohol tolerance, while an increased expression of the δ subunit could facilitate increased alcohol intake and withdrawal, creating distinct experimental conditions from naïve animals. Therefore, the complex changes that occur after longer times of exposure need to be taken into consideration when evaluating the effect of acute ethanol application on GABAergic transmission. The large data dispersion suggest that ethanol actions on GABA_A_R are complex and depend on still unidentified factors.

## Data that Support the Acute Action of Ethanol on GABA_A_Rs

### Ethanol Potentiates GABA_A_Rs in a Complex Manner

After the original report of GABA-mediated neurotransmission modulation by ethanol in the cat brain, several ligand binding studies detected small effects of ethanol on GABA_A_R (Nestoros, 1980a,b; Ticku and Burch, 1980; Ticku et al., 1983; Ticku, 1989). Functional analysis examining ^36^Cl^−^ efflux from synaptoneurosome preparations revealed potentiating effects of ethanol concentrations as low as 10 mM (Suzdak et al., 1986a,b; Allan and Harris, 1987; Mehta and Ticku, 1988; Harris, 1990; Engblom and Akerman, 1991). However, electrophysiological studies in brain slices and cultured neurons resulted in a considerable body of differing results, reporting ethanol potentiation in some brain areas, but not in others (Gage and Robertson, 1985; Barker et al., 1987; Siggins et al., 1987; White et al., 1990; Reynolds et al., 1992; Lovinger and Homanics, 2007). The first demonstration that ethanol caused a potentiation of GABA-induced Cl^−^ current was in hippocampal and cortical neurons using whole cell patch clamp recordings (Aguayo, 1991). This study was able to show that ethanol affected some cells, but not others in the same group of neurons. Also, it showed that the effect was independent from that produced by diazepam and barbiturates and that ethanol was unable to directly gate the ion channel. Other studies have largely confirmed this neuronal variability using cultured and acutely dissociated Purkinje cells, supporting the idea of a large complexity in this phenomenon (Sapp and Yeh, 1998; Criswell et al., 2003, 2008). Opposing results were also found between species. For example, GABA_A_R in mouse hippocampus were about 300 times more sensitive to ethanol than those in rats (Aguayo et al., 1994). Furthermore, a number of studies, such as those showing that ethanol action was evident after sensitizing β-adrenergic receptor pathways, suggested that intracellular signaling played a role in the modulation of ethanol sensitivity (Lin et al., 1994; Freund and Palmer, 1997; Yang et al., 1998). Ethanol sensitivity may also change throughout development, similar to the sensitivity to other drugs acting on GABA_A_R (Kapur and Macdonald, 1999; Aguayo et al., 2002).

Extensive studies of ethanol effects at concentrations between 0.1–850 mM in hippocampal neurons found three populations of neurons falling into different groups of sensitivity. The majority of neurons (60%) responded to moderate concentrations of ethanol with a maximal response at about 40 mM, a small group of cells (5%) was sensitive to concentrations below 1 mM, while in the remaining neurons only application of more than 100 mM showed an effect (Aguayo, 1991). These results support the existence of two separate mechanisms of ethanol action: (1) high and (2) low-to-moderate concentration effects on GABA_A_R, of which the second would be the relevant mode of action for conditions that are more likely to be encountered *in vivo* (Aguayo et al., 2002). Alternately, these results suggest a wide range of intracellular modulation of GABA_A_R sensitivity to ethanol. The study of native receptors in mammalian neurons found that currents less affected by ethanol were desensitizing and exhibited a significantly larger current run-down. Both of these properties are highly modulated by signal transduction pathways, suggesting that the difference between ethanol-sensitive and insensitive receptors may depend on differential intracellular modulation (Aguayo, 1990, 1991; Aguayo et al., 2002). Certain similarities between ethanol modulation and calcium regulation of GABA_A_R suggest that the effect of low to medium doses of ethanol is mediated by soluble intracellular components (Aguayo et al., 2002). Calcium and ethanol modulation of GABA_A_R vary between whole-cell and cell-free outside-out patch modes (Mozrzymas and Cherubini, 1998; Aguayo et al., 2002). An increase in intracellular Ca^2+^ elicits a higher ethanol sensitivity in some cell types and brain regions, while in others it has no effect on ethanol sensitivity (Taleb et al., 1987; Llano et al., 1991; Kellenberger et al., 1992; Aguayo et al., 1994). Both effects also display a similar concentration-response curve that demonstrates a potentiation of receptor function at intermediate concentrations, but inhibition at higher concentrations (Llano et al., 1991; Mouginot et al., 1991; Aguayo et al., 1994). The finding that potentiation of GABA_A_R currents at a low GABA and intermediate ethanol concentration fades over time, and ethanol response is reversibly lost after repeated ethanol pulses within a short time span, further supports the involvement of intracellular signaling (Aguayo et al., 2002).

### Post-Transcriptional Mechanisms Can Partly Explain the Variability of the Results

pLGICs are not the only proteins with a central role in the effects of ethanol on the brain. Several proteins involved in post-translational modification have also been reported as critical and might influence these receptors in a modulatory fashion. For instance, protein kinase C (PKC) activity has been associated with ethanol induced changes *in vitro*. Indeed, PKCε null mutant mice showed increased locomotion with low doses of ethanol and increased sedation with high doses of ethanol, while voluntary ethanol consumption was reduced (Hodge et al., 1999; Olive et al., 2000; Ueno et al., 2001). Interestingly, ethanol and flunitrazepam potentiation of GABA_A_R response to the direct agonist muscimol was increased in PKCε null mutants. Furthermore, treatment with a PKCε inhibitor mimicked the muscimol response of the PKCε null mutant in wild type animals, but had no additional effect in the mutant (Hodge et al., 1999). This change could be due to the lack of GABA_A_R γ2 phosphorylation at serine residue S327, which reduces the effect of benzodiazepines and ethanol on γ2 containing receptors (Qi et al., 2007). It would be an intriguing experiment to examine the effects of a phospho-mimetic or phosphorylation resistant GABA_A_R γ2 KI on the behavioral response to ethanol. This type of approach could help to determine which of the changes reported in PKCε null mutants rely on GABA_A_R γ2 S327 phosphorylation. KO of PKCδ resulted in an increased resistance to ethanol intoxication (Choi et al., 2008). PKCγ KO mice exhibited shorter loss of righting reflex and were less sensitive to ethanol-induced hypothermia (Harris et al., 1995). Also, there are several studies reporting that the sensitivity of GABA_A_R to ethanol can be affected by the activation of PKC, as well as signaling proteins such as G proteins (Weiner et al., 1997; Aguayo et al., 2002).

It is well accepted that protein kinases are able to modify GABA_A_R function, although the direction of this change, potentiating or depressing, depends not only on the type of kinase, but also on the neuron-type (Aguayo et al., 2002). The intracellular domain of the GABA_A_R γ2L subunit features a consensus sequence for PKC phosphorylation and it was reported that mutation of this region abolished PKC-dependent ethanol effects (Wafford et al., 1991; Wafford and Whiting, 1992). Contrary to this, phosphorylation of γ2 at residue S327 reduced the effect of benzodiazepines and ethanol on γ2 containing receptors (Qi et al., 2007). The differential influence of PKC on the action of ethanol was also reported in cultured hippocampal mouse neurons, where PKC activation reduced ethanol sensitivity and in rat hippocampal slices, where the activation of the kinase increased the effect of ethanol (Aguayo et al., 1994; Weiner et al., 1994, 1997). Interestingly, PKA activation was reported to positively influence ethanol sensitivity in cerebellar Purkinje neurons (Freund and Palmer, 1997). Furthermore, it appears that PKCε can facilitate alcohol-stimulated GABA release and regulate alcohol sensitivity of GABA_A_R (Olive et al., 2000; Qi et al., 2007; Bajo et al., 2008). Also, PKCδ may regulate the ethanol sensitivity of tonic GABAergic currents while PKCγ mediates ethanol-induced internalization of α1 containing GABA_A_R and increases the number of synaptic α4 containing receptors (Liang et al., 2007; Werner et al., 2011; Kumar et al., 2012). In addition, acute ethanol exposure also rapidly promotes the internalization of extra-synaptic GABA_A_R featuring the α4 and δ subunits (Liang et al., 2007; Shen et al., 2011).

### Effects of Ethanol on GABA_A_R Depend on Synaptic or Non-Synaptic Location

While it appears that physiological concentrations of alcohol between 1 and 100 mM selectively potentiate some GABA_A_R conformations, it has proven highly difficult to unquestionably identify the relevant subunits composing the sensitive receptors (for review see Aguayo et al., 2002; Lovinger and Homanics, 2007). Also, another level of complication arises from the possibility that ethanol sensitivity might rely on post-transcriptional modifications and receptor phosphorylation (Aguayo, 1990, 1991; Mozrzymas and Cherubini, 1998; Aguayo et al., 2002). Despite these constraints, there is a wide consensus that synaptic GABA_A_R are only potentiated by relatively high doses of 100 mM ethanol (Aguayo et al., 2002; Cui et al., 2012; Roberto et al., 2012). Thus, ethanol might differently affect synaptic and extra-synaptic GABA_A_R and in turn cause complex effects on phasic and tonic inhibition. Moreover, acute ethanol triggers several GABA_A_R independent effects in neuronal tissue modulating GABAergic transmission by a combination of pre- and postsynaptic actions (Lovinger and Homanics, 2007; Fleming et al., 2009). Potential cross talk mechanisms might exist between pre- and post-synaptic actions of ethanol. For example, a study showed that ethanol-sensitivity of GABA_A_R in hippocampal pyramidal cells was significantly altered after blocking GABA_B_R (Wan et al., 1996). These results are interesting since GABA_B_R are associated to G proteins and they could add another degree of complexity to understand how ethanol affects GABA_A_R.

It is widely accepted that anesthetic concentrations of ethanol (50–100 mM) can increase the probability of GABA release via pre synaptic mechanisms (Roberto et al., 2003; Carta et al., 2004; Nie et al., 2004; Kelm et al., 2011). Thus, tonic currents can be enhanced by ethanol affecting action potential-dependent spill-over of GABA from synaptic sites as demonstrated in cerebellar granule cells (Carta et al., 2004). These results, however, cannot explain the lack of effect of 30 mM on the tonic current recorded in hippocampal and thalamic neurons of PKCδ KO mice (Choi et al., 2008; see Table 3).

In cerebellar slices, acute ethanol exposure was shown not only to increase the frequency of spontaneous inhibitory postsynaptic currents mediated by synaptic GABA_A_R, but also to potentiate tonic currents mediated by extra-synaptic GABA_A_R and in granule cells (for review see Cui et al., 2012). The tonic currents in cerebellar granule cells are mediated by GABA_A_R containing α6 and δ subunits associated with either β2 or β3 subunits (Pöltl et al., 2003). Therefore, these findings lend support to previous studies that showed ethanol potentiation of recombinant receptors with similar subunit conformation. Wallner and collaborators found potentiation of Cl^−^ currents by concentrations of ethanol as low as 3 mM in Xenopus oocytes expressing recombinant GABA_A_R α6β3δ subunits (Wallner et al., 2003). Like most studies on the effect of ethanol on GABA_A_R, other laboratories have been unable to produce similar results. For example, additional studies with α6β3δ recombinant receptors in Xenopus oocytes failed to observe an effect of ethanol at concentrations between 7.5–30 mM (Baur et al., 2009). Moreover, studies in CHO cells expressing α6β3δ recombinant receptors reported no potentiating effect of 100 mM ethanol (Yamashita et al., 2006). More recent publications provided another plausible explanation for these conflicting findings by suggesting that the incorporation of the δ subunit into recombinant GABA_A_R is very dependent on DNA ratio and time of exposure (Meera et al., 2010). A concatenated receptor-construct fixing the GABA_A_R in the α6β3δ conformation was insensitive to ethanol as well (Baur et al., 2009). Furthermore, subunit compositions of extra-synaptic receptor populations at least in some investigated neuronal cell types, i.e., cerebellar granule neurons, seem to not be homogenous (Jechlinger et al., 1998; Sigel and Baur, 2000; Pöltl et al., 2003; Hanchar et al., 2005; Wallner et al., 2006). Taken together, these studies indicate that the elucidation of ethanol effects on GABA_A_R is far from resolved and more research is needed.

### Final Remarks on the Effects of Ethanol on GABA_A_Rs

The large volume of data collected in search of the mechanisms involved in the potentiation of GABA_A_R by ethanol has allowed us to view a detailed, but far from complete picture. Considering the body of controversial evidence, we have to conclude that there is still no agreement on what makes a given conformation of GABA_A_R sensitive to physiologically relevant concentrations of ethanol. The large number of receptor subunits and possible combinations thereof, as well as the differences observed in various brain areas and cell types, together with conflicting results make it hard to comprehend the mechanism of acute ethanol action on the receptor. The important role of receptor phosphorylation, principally by PKC, and possibly other posttranscriptional modifications and intracellular signaling mechanisms, such as the activation state of G protein, further convolute the story. Changes in the GABAergic system after chronic alcohol exposure, as well as indirect effects of alcohol like increased GABA release, also make the interpretation of results difficult. While the potentiation of the GABA_A_R at a high ethanol concentration is widely accepted and reproducible, the effect of low concentrations has remained elusive and no convincing mechanism which is able to explain the many conflicting results has emerged. A direct action of ethanol on the receptor alone seems insufficient to explain this potentiation and the question whether ethanol potentiates GABA_A_R by direct binding or by intracellular signaling mechanisms remains largely unresolved. It is possible that both mechanisms exist in parallel, although they might not have the same relevance for low and high concentrations of ethanol.

## GlyR as an Alternative Inhibitory Model of Study and Therapeutic Target

The existence of multiple potential conformations of GABA_A_R shifted the attention to another much simpler inhibitory member of the pLGIC family, the glycine receptor (GlyR). After the first demonstration that ethanol potentiates glycinergic Cl^−^ current in central mammalian neurons, there is now universal agreement that GlyR is an important target for low ethanol concentrations (Aguayo et al., 1994; Burgos et al., 2015). Several physiological and pharmacological properties are shared between GlyR and GABA_A_R. Both receptor channels increase membrane permeability to anions, primarily chloride ions, leading to a fast and potent inhibition of neuronal firing (Lester et al., 2004; Miller and Smart, 2010). They share the overall pentameric architecture and function typical for all members of the family. In contrast to GABA_A_R which are predominantly expressed in the brain, GlyR are the main inhibitory receptors in the mammalian spinal cord and brain stem, although they are also found in the hippocampus and other brain regions both pre- and postsynaptically (Eichler and Meier, 2008; Eichler et al., 2008, 2009; Winkelmann et al., 2014). It has been described that 10 mM ethanol potentiates glycinergic currents in cultured spinal neurons by increasing the apparent affinity of GlyR, without changing the efficacy of the NT (Aguayo et al., 1996; Mihic et al., 1997; Crawford et al., 2007; Perkins et al., 2008). In a synaptic context, ethanol also positively modulates glycinergic synaptic events increasing inhibitory effects in mature neurons (Eggers and Berger, 2004). Subsequent studies have shown that the ability of physiological concentrations of ethanol below 100 mM to potentiate GlyR depends on the receptor conformation, demonstrating different responses according to subunit composition. Unlike GABA_A_R, the case of GlyR is simpler and it has been confirmed that the α1 subunit is essential for the effects observed in the presence of ethanol (Sebe et al., 2003; Aguayo et al., 2004).

The GlyR is a promising candidate for pharmacological intervention against the effects of alcohol in the CNS for various reasons. There are five genes encoding for GlyR subunits, only four of which are relevant for the formation of functional receptor channels in humans. GlyR α subunits can form functional homomeric receptors and heteromeric receptors are generally composed of the β subunit and only one of the α subunits giving rise to only six possible receptor conformations. While they are not as prevalent in supratentorial regions as GABA_A_R, GlyR (including the ethanol sensitive α1 subunit) have been reported in several regions of the brain. In fact, it has recently been shown that in neurons of the lateral orbitofrontal cortex, ethanol inhibition relies mostly on GlyR (Badanich et al., 2013). Also, there are far less conflicting results regarding GlyR and low concentrations of ethanol (5–10 mM), and the mechanism of action is better understood.

Similar to GABA_A_R, one proposed mechanism involves the interaction of ethanol with a group of amino acids, including alanine 52 (N-terminal), serine 267 (TM2) and alanine 288 (TM3), that form a binding pocket for direct ethanol interaction. Mutagenesis studies demonstrated loss of ethanol induced GlyR potentiation by alteration of the critical amino acid residues and more recently, the model has been refined by structural studies (Blednov et al., 2012; Howard et al., 2014; Trudell et al., 2014). As discussed for GABA_A_R, however, loss of an ethanol sensitive phenotype may not be strong enough evidence to support the contribution of a direct ethanol-binding site involved in the mechanism of potentiation. The three residues suggested to be involved in ethanol binding are likely critical for channel gating after ligand binding. The evidence suggests the existence of a hydrophobic binding pocket for general anesthetics and alcohol formed by the residues previously described. This site might be responsible for the potentiation of GlyR by ethanol, especially at higher concentrations. However, the lack of pharmacological specificity of this site and the dramatic alterations in physiological properties of the channel caused by the S267 mutation including desensitization, changes in apparent affinity, and pharmacological selectivity complicates the interpretation of these results (Lobo et al., 2004; Sine and Engel, 2006). Another alternative mechanism is based on the findings that the effect of ethanol on native and recombinant GlyR is blocked by GDP-β-S, sequestration of Gβγ using Gβγ-specific antibodies, Gα overexpression, and peptides that bind to Gβγ with high affinity (Burgos et al., 2015). In addition, mutant receptors that have reduced binding capacity to Gβγ are much less sensitive to low ethanol concentrations (Yevenes et al., 2008; Burgos et al., 2015). All of these studies support the idea that ethanol effects on GlyR are dependent on Gβγ activation. This hypothesis is supported by recent studies using a KI mouse model with mutations in the intracellular loop of the receptor. These animals displayed glycinergic currents insensitive to ethanol and Gβγ (Aguayo et al., 2014). The sedative effect of ethanol in these animals was likewise reduced (Burgos et al., 2015). These results indicate that ethanol can potentiate GlyR at low concentrations via G-protein interaction (Aguayo et al., 1996). According to this model, Gβγ binds to the large intracellular loop of the receptor, and the interaction can be interfered with by small molecules (Guzman et al., 2009). This indirect mode of ethanol action may more readily give rise to pharmacological intervention with higher specificity and fewer unwanted effects than interference with ethanol binding at the TM domain of the GABA_A_R. Moreover, further investigation on the mechanism of action of low ethanol concentrations on GlyR may help to elucidate the molecular mechanisms involved in the modulation of GABA_A_R by ethanol. Therefore, the GlyR could serve as a valuable model to learn about the effects of alcohol on the two most prevalent mediators of inhibition in the CNS with the aim of developing new pharmacological tools. Before this is accomplished, it will be most critical to determine the role of supratentorial GlyRs on the effects of ethanol, to identify the more abundant receptor conformation in key brain regions, and develop subunit specific ligands to modulate the actions of ethanol on these targets.

## Author Contributions

BF contributed to all stages of manuscript preparation and editing. PAC and GM-C contributed with critical discussions and figure design. LGA contributed to all stages of manuscript preparation and final editing.

## Conflict of Interest Statement

The authors declare that the research was conducted in the absence of any commercial or financial relationships that could be construed as a potential conflict of interest.
